# LC and NMR Studies for Identification and Characterization of Degradation Byproducts of Olmesartan Acid, Elucidation of Their Degradation Pathway and Ecotoxicity Assessment

**DOI:** 10.3390/molecules26061769

**Published:** 2021-03-22

**Authors:** Giovanni Luongo, Antonietta Siciliano, Giovanni Libralato, Sara Serafini, Lorenzo Saviano, Lucio Previtera, Giovanni Di Fabio, Armando Zarrelli

**Affiliations:** 1 Department of Chemical Sciences, University of Naples Federico II, 80126 Naples, Italy; giovanni.luongo@unina.it (G.L.); difabio@unina.it (G.D.F.); 2 Department of Biology, University of Naples Federico II, 80126 Naples, Italy; antonietta.siciliano@unina.it (A.S.); giovanni.libralato@unina.it (G.L.); sara.serafini@unina.it (S.S.); lorenzosaviano@libero.it (L.S.); 3 Associazione Italiana per la Promozione delle Ricerche su Ambiente e Salute Umana, 82030 Dugenta, Italy; previter@unina.it

**Keywords:** olmesartan acid, chlorination, hypochlorite, degradation byproducts, water treatment, *Aliivibrio fischeri*, *Raphidocelis subcapitata*

## Abstract

The discovery of various sartans, which are among the most used antihypertensive drugs in the world, is increasingly frequent not only in wastewater but also in surface water and, in some cases, even in drinking or groundwater. In this paper, the degradation pathway of olmesartan acid, one of the most used sartans, was investigated by simulating the chlorination process normally used in a wastewater treatment plant to reduce similar emerging pollutants. The structures of nine isolated degradation byproducts (DPs), eight of which were isolated for the first time, were separated via chromatography column and HPLC methods, identified by combining nuclear magnetic resonance and mass spectrometry, and justified by a proposed mechanism of formation beginning from the parent drug. Ecotoxicity tests on olmesartan acid and its nine DPs showed that 50% of the investigated byproducts inhibited the target species *Aliivibrio fischeri* and *Raphidocelis subcapitata*, causing functional decreases of 18% and 53%, respectively.

## 1. Introduction

Keeping water clean and healthy is difficult, but not impossible. The important thing is to define which substances actually constitute a danger and a risk to the health of humans and other species, to establish which ones must leave production cycles to be eliminated from the environment, and to determine which ones must be kept in concentrations below certain hazardous limits. In water, new substances, in addition to being highly persistent contaminants, carry possible health and environmental effects. Pollutants of emerging interest are considered one of the most significant environmental problems in recent years. These include perfluoroalkyl substances (PFAS) [1], cyanobacteria [2], mycotoxins [3], hormones [4], psychoactive substances [5], pesticides [6], cosmetics, and industrial additives and drugs [7]. These substances have the potential to cause adverse effects on the environment and human health, but are still largely not specifically regulated by legislation, and their effects are not yet clear [8,9,10,11,12,13,14,15]. At the local level, pollution could affect different types of substances—and also byproducts or reaction products—so much so that an aggregate and cumulative risk assessment is required that takes into account the multiplicity of exposures. The effluents of wastewater treatment plants (WWTPs), not normally designed to eliminate emerging contaminants, are one of the main sources of contamination of surface waters by micropollutants [16]. Hundreds of tons of pharmaceutical substances flow to WWTPs every year, hence the need to implement WWTPs with advanced treatments, tertiary wastewater treatments, capable of improving removal efficiencies. Advanced wastewater treatment processes are the subject of numerous studies. Technologies such as ozonation [17,18,19], membrane filtration [20], adsorption [21] and above all advanced oxidation [22], are configured as systems capable of improving the removal of emerging contaminants from wastewater.

Sartans, among the most marketed antihypertensive drugs in the world, are now considered ubiquitous emerging contaminants in many countries [23]. In particular, olmesartan acid (OLM) is one of these [24]. It is marketed as olmesartan medoximil, the prodrug from which the active pharmaceutical form, olmesartan acid, is released in the intestinal mucosa and rapidly enters the bloodstream [25,26]. However, the olmesartan acid is released as-is through feces and urine. In recent studies, carried out in some European countries, it was found that the concentration of olmesartan acid in wastewater reaches up to 1200 ng/L [23], but in surface waters ranges from 150 ng/L to 800 ng/L, with peaks above 2 μg/L in some German rivers [24]. However, the concentrations are lower in groundwater [27].

The advanced oxidation processes while being effective in the removal of micropollutants, if not carried out properly, can lead to the formation of intermediate reaction products, often more toxic than the starting compounds [28,29,30,31,32,33].

In this paper, the degradation byproducts (DPs) of OLM were investigated under the same conditions as the chlorination process normally used in WWTPs to reduce similar emerging pollutants [24,29], carrying out two different experiments, one at concentrations comparable to those at which OLM is present in the wastewaters (10^−5^ M) and one at concentrations at least 100 times higher in order to isolate and identify the DPs. The structures of 9 isolated DPs, eight of which were isolated for the first time, have been determined by combining nuclear magnetic resonance (NMR) and mass spectrometry (MS), using matrix-assisted laser desorption/ionization as a source and a time-of-flight analyzer (MALDI-TOF) for mass analysis. These methods were justified by a proposed mechanism of formation.

The evaluation of OLM degradation and the formation of by-products was subjected to ecotoxicological tests performed with *Aliivibrio fischeri* bacteria and *Raphidocelis subcapitata* algae. In order to monitor the performance of OLM degradation, the ecotoxicological assays represent an advantageous technique for providing information about environmental risk regarding treated effluent discharge or even in subsequent/tertiary treatments.

## 2. Materials and Methods

### 2.1. Drug and Reagents

Olmesartan acid (99.5%) was purchased from Sigma Aldrich (Milan, Italy). All the other chemicals and solvents were purchased from Fluka (Saint-Quentin Fallavier, France) and were of HPLC grade and used as received. For the antimicrobial assessment, tryptic soy broth (TSB, Difco, Becton-Dickenson Labs) was used. All chemicals were of analytical grade and supplied by Sigma Aldrich. Double-distilled water (Microtech) was used to prepare dilution water and the treatments. Microbial growth was measured with an automatic plate reader (Synergy HTX, BioTek Instruments, Winooski, VT, USA).

### 2.2. Chlorination Reaction

#### 2.2.1. Apparatus and Equipment

Column chromatography (CC) was carried out with Kieselgel 60 (230–400 mesh, Merck, Darmstadt, Germany). HPLC was performed on a Shimadzu LC-8A system using a Shimadzu SPD-10A VP UV-VIS detector (Shimadzu, Milan, Italy). Preparative HPLC was performed using an RP Gemini C18-110A preparative column (10 μm particle size, 250 × 21.2 mm i.d., Phenomenex, Bologna, Italy) with a flow rate of 7.0 mL/min. The ^1^H- and ^13^C-NMR spectra were recorded with an NMR spectrometer operated at 400 MHz and at 25 °C (Bruker DRX, Bruker Avance, Billica, MA, USA) and referenced in ppm to the residual solvent signals (CDCl_3_, at δ_H_ 7.27 and δ_C_ 77.0). The proton-detected heteronuclear correlations were measured using a gradient heteronuclear single-quantum coherence (HSQC) experiment, optimized for ^1^J_HC_ = 155 Hz, and a gradient heteronuclear multiple bond coherence (HMBC) experiment, optimized for ^n^J_HC_ = 8 Hz. The MALDI TOF mass spectrometric analyses were performed on a Voyager-De Pro MALDI mass-spectrometer (PerSeptive Biosystems, Framingham, MA, USA).

#### 2.2.2. Chlorination Experiments

**Scheme 1:**
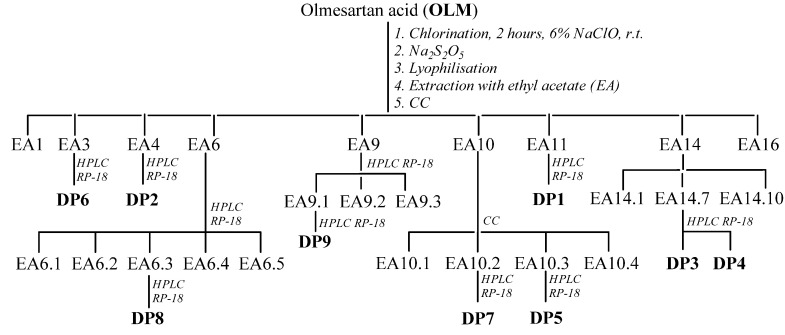
The isolation of the different identified compounds.

**Figure 1:**
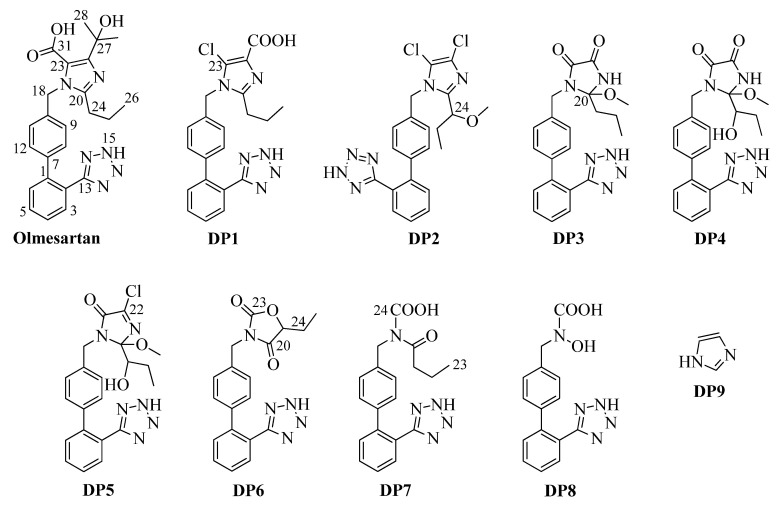
Chemical structures of olmesartan acid and its degradation byproducts DP1–DP9.

A 10^−5^ M OLM solution was treated for 10 min with 10% hypochlorite (molar ratio OLM/HClO 1:1 concentration, spectroscopically determined λ_max_ 292 nm, ε 350 dm^3^/mol cm) at room temperature [34], simulating the conditions used in a typical WWTP. The experiment was conducted at pH = 10.5. The presence of OLM was quantified using a Lambda 12 UV-Vis spectrophotometer (Perkin Elmer, Waltham, MA, USA). Absorbance peaks were determined at 230 nm. The absorbance values were converted into a concentration using a calibration curve prepared from standard solutions with known OLM concentrations. The pH of the solution, measured and recorded continuously using a pH-meter, increased immediately from the initial pH of 8.0 to 10.5, and pH remained at this value during the reaction. An aliquot of the solution was taken every 15 min, quenched by sodium thiosulphate excess, filtered, dried by lyophilization, and the residue dissolved in a saturated sodium bicarbonate solution and extracted with ethyl acetate. The course of the reaction was monitored using HPLC. DP1–DP9 were isolated from the ethyl acetate extract of the aqueous solution (Scheme 1 and Figure 1) and identified by comparing their retention times with those of commercially available standard compounds, or isolated by performing preparative experiments with a solution of OLM at a concentration higher than 10^−3^ M and treated with 6% hypochlorite at room temperature for 2 h. The DPs obtained were isolated using column chromatography and HPLC and were completely characterized using NMR and MS analyses.

#### 2.2.3. Chlorination Procedure and Product Isolation

Olmesartan acid (1.0 g, 2.24 mmol) was dissolved in 1.0 L of phosphate buffer (KH_2_PO_4_/K_2_HPO_4_ 0.1 M) [35]. A sodium hypochlorite solution (about 6% active chlorine, molar ratio OLM/HClO 1:20; concentration spectroscopically determined at λ_max_ of 292 nm, ε = 350 dm^3^/mol × cm) was added drop-by-drop to this solution under magnetic stirring at room temperature. The pH of phosphate buffer was adjusted at a value of 6.50 by adding a 10% H_3_PO_4_ solution, checked with a pH-meter. The reaction was stopped after 2 h with an excess of sodium thiosulphate and concentrated by lyophilization. The residue was dissolved in water and pH adjusted to 7.00, and this solution was extracted using ethyl acetate (EA). The crude EA fraction (579 mg) was chromatographed on silica gel CC, eluting with gradient of methylene chloride:methanol:acetic acid (100:0:0.5 to 70:30:0.5, *v*/*v*/*v*) to yield 16 fractions. The fraction EA3 (16 mg), eluted with methylene chloride/methanol/acetic acid (100:0:0.5, *v*/*v*/*v*), was analyzed via HPLC using a reversed-phase column and eluting with water/acetonitrile (20:80, *v*/*v*) to yield DP6 (6.7 mg). The fraction EA4 (30 mg), eluted with methylene chloride:methanol:acetic acid (98:2:0.5, *v*/*v*/*v*), was separated by semipreparative HPLC using a reversed-phase column Kromasil 10 µm 100 Å C18 (250 × 10 mm) and elution with a gradient of CH_3_COONH_4_ (A, pH 4.0; 10 mM) and methanol (B), starting with 70% B for 1 min and installing a gradient to obtain 100% B over 20 min at a solvent flow rate of 4 mL/min to yield DP2 (7.8 mg). The fraction EA6 (36 mg), eluted with methylene chloride:methanol:acetic acid (95:5:0.5, *v*/*v*/*v*), was analyzed via preparative HPLC using a reversed-phase column Gemini 10 µm C18 110 Å (250 × 21 mm) and elution with a gradient of CH_3_COONH_4_ (A, pH 4.0; 10 mM) and methanol (B), starting with 60% B for 1 min and installing a gradient to obtain 100% B over 30 min at a solvent flow rate of 7.5 mL/min to yield 5 subfractions.

The subfraction EA6.3 (7 mg) was re-chromatographed via HPLC using a reversed-phase column Luna 5 µm 100 Å C18(2) (150 × 4.6 mm) and elution with a gradient of CH_3_COONH_4_ (A, pH 4.0; 10 mM) and acetonitrile (B), starting with 60% B for 1 min and installing a gradient to obtain 100% B over 30 min at a solvent flow rate of 1 mL/min to yield DP8 (1.3 mg). The fraction EA9 (13 mg), eluted with methylene chloride:methanol:acetic acid (90:10:0.5, *v*/*v*/*v*) was separated via preparative HPLC using a reversed-phase column Gemini 10 µm C18 110 Å (250 × 21 mm) and elution with a gradient of CH_3_COONH_4_ (A, pH 4.0; 10 mM) and methanol (B), starting with 50% B for 1 min and installing a gradient to obtain 100% B over 30 min at a solvent flow rate of 7.5 mL/min to yield 3 subfractions. The subfraction EA9.1 (15 mg) was re-chromatographed via HPLC using a reversed-phase column Kinetex 2.6 µm 100 Å C18 (100 × 4.6 mm) and elution with a gradient of CH_3_COONH_4_ (A, pH 4.0; 10 mM) and acetonitrile (B), starting with 40% B for 1 min and installing a gradient to obtain 100% B over 30 min at a solvent flow rate of 0.8 mL/min to yield DP9 (0.9 mg). The fraction EA10 (30 mg), eluted with methylene chloride:methanol:acetic acid (85:15:0.5, *v*/*v*/*v*), was analyzed via semipreparative HPLC using a reversed-phase column Kromasil 10 µm 100 Å C18 (250 × 10 mm) and elution with a gradient of CH_3_COONH_4_ (A, pH 4.0; 10 mM) and methanol (B), starting with 35% B for 1 min and installing a gradient to obtain 100% B over 20 min at a solvent flow rate of 4 mL/min to yield 4 subfractions. The subfractions EA10.2 (7 mg) and EA10.3 (9 mg) were re-chromatographed by HPLC using a reversed-phase column Kinetex 2.6 µm 100 Å C18 (100 × 4.6 mm) and elution with a gradient of acetic acid/acetonitrile (A, 1:99, *v*/*v*) and acetic acid/water (B, 1:99, *v*/*v*), starting with 75% B for 3 min and installing a gradient to obtain 100% A over 35 min and returning to 75% B for 10 min, at a solvent flow rate of 0.8 mL/min. They contained DP7 (2.6 mg) and DP5 (5.9 mg), respectively. The fraction EA11 (63 mg), eluted with methylene chloride:methanol:acetic acid (80:20:0.5, *v*/*v*/*v*), was analyzed via preparative HPLC using a reversed-phase column Gemini 10 µm C18 110 Å (250 × 21 mm) and elution with a gradient of CH_3_COONH_4_ (A, pH 4.0; 10 mM)) and acetonitrile (B), starting with 20% B for 1 min and installing a gradient to obtain 90% B over 25 min at a solvent flow rate of 8 mL/min to yield DP1 (7.1 mg). The fraction EA14 (120 mg), eluted with methylene chloride:methanol:acetic acid (70:30:0.5, *v*/*v*/*v*), was re-chromatographed on silica gel CC by elution with a gradient of chloroform:acetone:acetic acid (100:0:0.5 to 60:40:0.5, *v*/*v*/*v*) to yield 10 subfractions. The subfraction EA14.7 (33 mg), eluted with chloroform:acetone:acetic acid (70:30:0.5, *v*/*v*/*v*), was separated by semipreparative HPLC using a reversed phase column Kromasil 10 µm 100 Å C18 (250 × 10 mm) and elution with a gradient of CH_3_COONH_4_ (A, pH 4.0; 10 mM) and methanol (B), starting with 0% B for 5 min and installing a gradient to obtain 100% B over 20 min at a solvent flow rate of 3.5 mL/min to yield DP3 (11.6 mg) and DP4 (6.6 mg).

### 2.3. Ecotoxicity Data

The acute bioluminescence test with *Aliivibrio fischeri* (NRRLB-11177) was carried out in accordance with the ISO 11348-3:2007 [36] standard protocol, and the algal growth inhibition test with *Raphidocelis subcapitata* was performed according to the ISO 8692:2012 [37] standard protocol. These organisms are established biological models and are included in most regulations for the assessment of wastewater on an end-of-pipe basis.

The inhibition of bioluminescence in the presence of the OLM and its DPs was measured after 30 min of exposure. The toxic effect values are given by the ratio of the decrease in bacterial light output emitted by the bacterium in the sample compared to the control. To provide the relevant osmotic pressure for the test organisms, the salinity concentration of the stock solution was adjusted by 2% for NaCl. The temperature during exposure was 15 °C according to the Microtox standard procedure.

The growth of algae exposed to the sample was compared with the growth of algae in a negative control. For each sample, three replicates were inoculated with 10^4^ algal cells L^−1^ in well plates and incubated for 72 h at 23 ± 2 °C under continuous illumination. The specific growth rate of *R. subcapitata* in each replicate was calculated from the logarithmic increase in cell density in the interval from 0 to 72 h. *R. subcapitata* density was determined by an indirect procedure using a spectrophotometer (Hach Lange DR5000, Loveland, CO, USA) and a 1 cm cuvette.

## 3. Results and Discussion

### 3.1. Chlorination Experiments

The OLM chlorination experiments were performed by mimicking the conditions of a typical WWTP, in which a 10^−5^ M solution of the drug was treated for 10 min with 10% hypochlorite (OLM:hypochlorite molar ratio of 1:1; concn.) at room temperature [38,39]. Then, the tests are repeated at much higher concentrations of the contaminant (>10^−3^ M), with a much lower ratio of OLM:oxidizing agent (1:5 or 1:6), so as to ensure the degradation of the studied contaminant and the possibility of isolating sufficient quantities of degradation byproducts for the subsequent structural identification.

**Figure 2:**
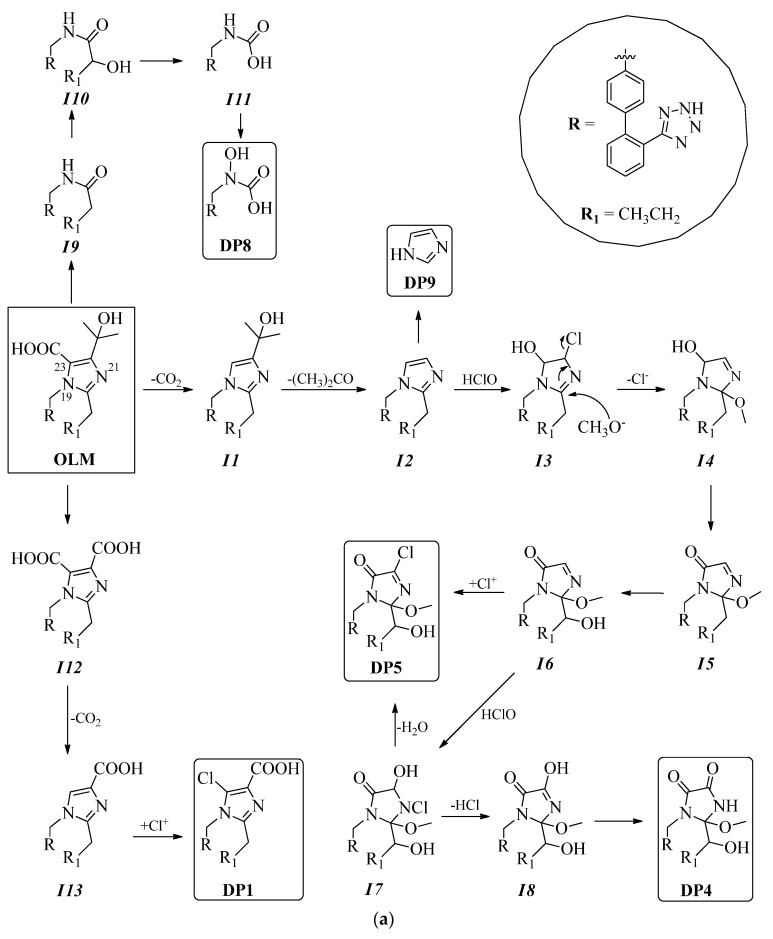

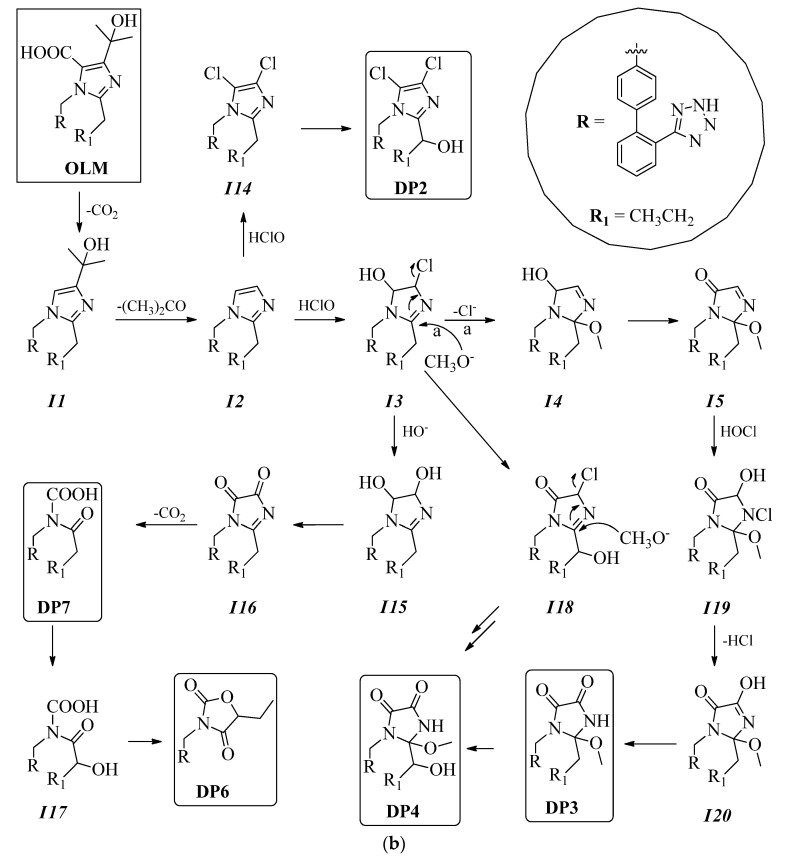
(**a**) Plausible mechanism for the formation of DP1, DP4, DP5, and DP8–DP9. (**b**) Plausible mechanism for the formation of DP2–DP4, DP6–DP7.

The course of the reaction was monitored by HPLC, and the DPs obtained were isolated by column chromatography and HPLC (Scheme 1) and completely characterized using NMR and MS analyses (see Supplementary Materials). Finally, DP1–DP9 (Figure 1) were isolated relative percentages of 1.23, 1.35, 2.01, 1.15, 1.02, 1.15, 0.45, 0.23, and 0.15, respectively. The proposed mechanism of their formation from OLM is shown in Figure 2a,b. Except for DP9, all other DPs were isolated for the first time.

### 3.2. Structure Elucidation of Degradation Byproducts DP1–DP9

In the OLM treatment at the buffered pH, the concentration of DP1–DP9 was at a maximum after 2 h and in the range of 2.01 to 0.15%. The plausible mechanism of the DPs formation from OLM is shown in Figure 2a,b. Olmesartan acid could undergo the oxidation of the side-chain at carbon C23 to obtain intermediate *I1,* whose subsequent decarboxylation at carbon C22 generates intermediate *I2*. The reaction of this with HClO could generate intermediate *I3* from which intermediate *I4* can be obtained via nucleophilic attack of the CH_3_O^−^ ion [40,41,42]. The oxidation at carbon C23 (intermediate *I5*) and C24 (intermediate *I6*) and the chlorination at carbon C22 could provide DP5. Intermediate *I6,* via its reaction with HClO, provides intermediate *I7,* which due to the loss of HCl would provide intermediate *I8.* From *I8,* the oxidation of DP4 could be obtained. Olmesartan acid could undergo the hydrolysis of the N19-C23 and C20-N21 bonds to release intermediate *I9*, from which intermediate *I10* could then be obtained via oxidation of the side-chain, precisely on the carbon adjacent to the carbonyl function. The complete oxidation of the *I10* side-chain would provide *I11,* and from the nitrogen oxidation of the latter, DP8 could be obtained. The partial oxidation of the C22 carbon of the starting product could provide intermediate *I12*, which via the decarboxylation at carbon C23 would provide intermediate *I13*. The chlorination of this could provide DP1. DP9 could be obtained from different precursors with the loss of all the substituents of the imidazole ring.

The intermediate *I2* could be chlorinated at carbons C22 and C23 to provide the intermediate *I14,* which could then provide DP2 via oxidation of the side-chain to carbon C24. Intermediate *I3,* by replacing the chlorine with OH^−^, could provide the intermediate *I15* which then, via the oxidation and opening of the imidazole ring, could provide DP7 through intermediate *I16*. DP7 could then provide intermediate *I17* via oxidation of the adjacent carbon of the carbonyl function of the side-chain, and then lactone DP6 could be obtained via its intramolecular reaction.

Intermediate *I5* could provide the corresponding *I19* by reaction with HClO. The loss of HCl (intermediate *I20*) and subsequent oxidation at carbon C22 could yield DP3. The oxidation of the latter to the C-24 carbon of the side chain could provide DP4.

### 3.3. Spectral Data

Olmesartan medoximil: (5-methyl-2-oxo-1,3-dioxol-4-yl)methyl 1-((2′-(2H-tetrazol-5-yl)-[1,1′-biphenyl]-4-yl)methyl)-4-(2-hydroxypropan-2-yl)-2-propyl-1H-imidazole-5-car boxylate. White powder. ^1^H- and ^13^C-NMR, see Table S1.

Olmesartan acid: *1-((2*′*-(2H-tetrazol-5-yl)-[1,1*′*-biphenyl]-4-yl)methyl)-4-(2-hydroxypropan-2-yl)-2-propyl-1H-imidazole-5-carboxylic acid.* ^1^H- and ^13^C-NMR, see [43]. MS-TOF (positive ions): m/z calculated for C_24_H_26_N_6_O_3_ *m*/*z* 446.21 [M]^+^; found 447.25 [M + H]^+^ (63%).

DP-1: *1-((2*′*-(2H-tetrazol-5-yl)-[1,1*′*-biphenyl]-4-yl)methyl)-5-chloro-2-propyl-1H*–*imidazo le-4-carboxylic acid*. White powder. ^1^H- and ^13^C-NMR, see Table S2. MS-TOF (positive ions): m/z calculated for C_21_H_19_ClN_6_O_2_ *m*/*z* 422.13 [M]^+^; found 426.16 [M + 3 + H]^+^ (6%), 425.16 [M + 2 + H]^+^ (30%), 424.16 [M + 1 + H]^+^ (15%), 423.15 [M + H]^+^ (90%).

DP-2: *5-(4*′*-((4,5-dichloro-2-(1-methoxypropyl)-1H-imidazol-1-yl)methyl)-[1,1*′*-biphenyl]-2-yl)-2H-tetrazole*. White powder. ^1^H- and ^13^C-NMR, see Table S3. MS-TOF (positive ions): m/z calculated for C_21_H_20_Cl_2_N_6_O *m*/*z* 442.11 [M]^+^; found 448.13 [M + 5 + H]^+^ (1%), 447.16 [M + 4 + H]^+^ (15%), 446.16 [M + 3 + H]^+^ (15%), 445.16 [M + 2 + H]^+^ (61%), 444.17 [M + 1 + H]^+^ (21%), 443.15 [M + H]^+^ (95%).

DP-3: *1-((2*′*-(2H-tetrazol-5-yl)-[1,1*′*-biphenyl]-4-yl)methyl)-2-methoxy-2-propyl imidazolidine-4,5-dione*. White powder. ^1^H- and ^13^C-NMR, see Table S4. MS-TOF (positive ions): m/z calculated for C_21_H_22_N_6_O_3_ *m*/*z* 406.18 [M]^+^; found 407.21 [M + H]^+^ (81%).

DP-4: *1-((2*′*-(2H-tetrazol-5-yl)-[1,1*′*-biphenyl]-4-yl)methyl)-2-(1-hydroxypropyl)-2-methoxyimidazolidine-4,5-dione*. White powder. ^1^H- and ^13^C-NMR, see Table S5. MS-TOF (positive ions): m/z calculated for C_21_H_22_N_6_O_4_ *m/z* 422.17 [M]^+^; found 423.20 [M + H]^+^ (73%).

DP-5: *3-((2*′*-(2H-tetrazol-5-yl)-[1,1*′*-biphenyl]-4-yl)methyl)-5-chloro-2-(1-hydroxypropyl)-2-methoxyimidazolidin-4-one.* White powder. ^1^H- and ^13^C-NMR, see Table S6. MS-TOF (positive ions): m/z calculated for C_21_H_23_ClN_6_O_3_ *m*/*z* 442.15 [M]^+^; found 443.19 [M + H]^+^ (80%), 445.18 [M + 2 + H]^+^ (25%).

DP-6: *3-((2*′*-(2H-Tetrazol-5-yl)-[1,1*′*-biphenyl]-4-yl)methyl)-5-ethyloxazolidine-2,4-dione.* White powder. ^1^H- and ^13^C-NMR, see Table S7. MS-TOF (positive ions): m/z calculated for C_19_H_17_N_5_O_3_ *m*/*z* 363.13 [M]^+^; found 364.18 [M + H]^+^ (71%).

DP-7: *((2*′*-(2H-Tetrazol-5-yl)-[1,1*′*-biphenyl]-4-yl)methyl)(butyryl)carbamic acid.* White powder. ^1^H- and ^13^C-NMR, see Table S8. MS-TOF (positive ions): m/z calculated for C_19_H_19_N_5_O_3_ *m*/*z* 365.15 [M]^+^; found 366.19 [M + H]^+^ (57%).

DP-8: *((2*′*-(2H-Tetrazol-5-yl)-[1,1*′*-biphenyl]-4-yl)methyl)(hydroxy)carbamic acid.* White powder. ^1^H and ^13^C-NMR, see Table S9. MS-TOF (positive ions): m/z calculated for C_15_H_13_N_5_O_3_ *m*/*z* 311.10 [M]^+^; found 312.14 [M + H]^+^ (29%).

DP-9: *1H-imidazole.* Identified by comparison with an authentic sample.

### 3.4. Ecotoxicity Data

**Figure 3:**
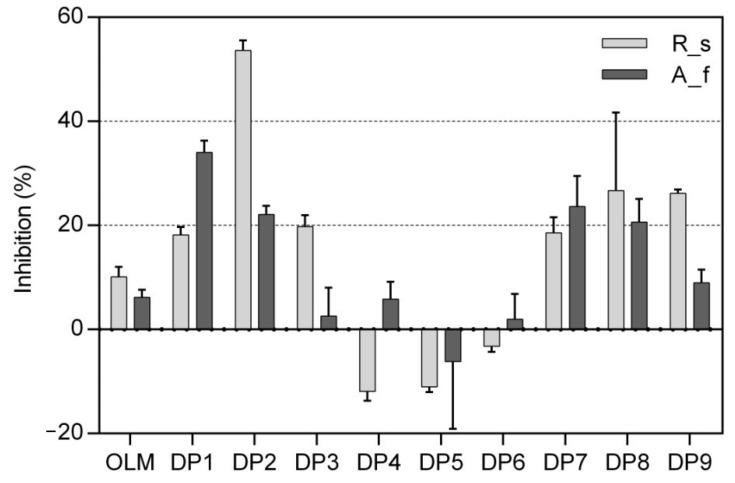
The toxicity data of olmesartan acid (OLM) and its byproducts (DP1–DP9) with 72 h *R. subcapitata* (R_s) and *A. fischeri* (A_f).

Toxicity data were summarized in Figure 3, including the effects of *R. subcapitata* 72 h and *A. fischeri* after 30 min. The analysis of toxicity data evidenced the presence of the three main groups of samples considering a threshold value for the effects statistically different from the control groups [44]: (i) no effect (−10% ≤ inhibition ≤ 10%); (ii) biostimulation effect (inhibition < −10%); and (iii) toxic effects (inhibition > 10%).

After 72 h of exposure, OLM presented no effect on *R. subcapitata*. Comparing OLM and DPs results, only DP6 showed no toxicity. Several samples presented negative growth effects (DP1, DP2, DP3, DP7, DP8, DP9), and their toxicity values ranged from 18% to a maximum of 53%. DP4 and DP5 showed stimulatory effects. Toxicity data from *A. fischeri* confirmed that the parent compound OLM showed no toxic effect after 30 min of exposure. Five of nine isolated DPs (DP3, DP4, DP5, DP6 and DP9) showed no inhibitory effect. The other DPs presented acute toxicity like DP1, DP2, DP7, and DP8 showing inhibition effects from 20% to 34%. Comparing the results of the algal growth inhibition with bacteria luminescence inhibition, it was evident that the response of *A. fischeri* was less sensitive, but the toxicity trends were linearly correlated (Pearson correlation, *r* = 0.61, moderately high correlation).

## 4. Conclusions

This paper investigated the fate of OLM following the degradation treatment by chlorination. The reaction was carried out by simulating the conditions of a typical WWTP using excess sodium hypochlorite. After the chlorination treatment, chromatographic techniques were used to isolate nine degradation byproducts, which were fully characterized by MS and NMR analyses and via comparison with a commercial standard. OLM underwent complete mineralization in about 59% of cases, and in 21% of cases was recovered as is. OLM transformed into the corresponding byproducts in 20% of cases, and about 9% of these were identified. A possible mechanism for the degradation of OLM and its degradation byproducts has been hypothesized. Half of the investigated DPs possessed anywhere from slightly to highly toxic effects on the target species *Aliivibrio fischeri* and *Raphidocelis subcapitata*; the remaining DPs presented no such effects. According to the selected battery of toxicity, the correlation of the results suggested that DPs acted very similarly in unicellular organisms. Moreover, due to the highlighted ecotoxicological effects, DPs acting on organisms of low levels of complexity could have negative effects also on the whole trophic chain (biomagnification).

## Data Availability

Not applicable.

## Supplementary Materials

The following are available online, Table S1: ^1^H, ^13^C and ^2^D NMR data of Olmesartan medoximil in CD 3 OD; Table S2: ^1^H, ^13^C and ^2^D NMR data of DP-1 in CD 3 OD; Table S3: ^1^H, ^13^C and ^2^D NMR data of DP-2 in CD 3 OD; Table S4: ^1^H, ^13^C and ^2^D NMR data of DP-3 in CD 3 OD; Table S5: ^1^H, ^13^C and ^2^D NMR data of DP-4 in CD 3 OD; Table S6: ^1^H, ^13^C and ^2^D NMR data of DP-5 in CD 3 OD; Table S7: ^1^H, ^13^C and ^2^D NMR data of DP-6 in CD 3 OD; Table S8: ^1^H, ^13^C and ^2^D NMR data of DP-7 in CD 3 OD; Table S9: ^1^H, ^13^C and ^2^D NMR data of DP-8 in CD 3 OD.

## Sample Availability

Samples of the compounds are not available from the authors.
