# Supplementary material for: LC and NMR Studies for Identification and Characterization of Degradation Byproducts of Olmesartan Acid, Elucidation of Their Degradation Pathway and Ecotoxicity Assessment
Source: Molecules. 2021 Mar 22;26(6):1769. doi: 10.3390/molecules26061769 (PMC8004111; doi:10.3390/molecules26061769)
Supplement: Supplementary file 1 [file molecules-26-01769-s001.pdf]

Supplementary Material

LC and NMR Studies for Identification and Characterization of Degradation Byproducts of Olmesartan, Elucidation of Their Degradation Pathway and Ecotoxicity Assessment

Giovanni Luongo, Antonietta Siciliano, Giovanni Libralato, Sara Serafini, Lorenzo Saviano, Lucio Previtera, Giovanni Di Fabio and Armando Zarrelli*

* Correspondence: zarrelli@unina.it; Tel.: +39-081-674-472

Table S1. [1] H, ^{13}C and 2D NMR data of Olmesartan medoximil in CD_3OD.

Position	Residue	$^{13}C^a$	$^1H^a$, multiplicity (J in Hz)	^1H-^1H COSY	^1H-^{13}C HMBC
1	C	142.99	-		7.66, 7.63, 7.58, 7.07
2	C	124.80	-		7.63, 7.58, 7.54
3	CH	131.78	7.63, d (7.2)	7.66, 7.54	7.66, 7.54
4	CH	129.02	7.54, dd (7.0, 1.3)	7.66, 7.63, 7.58	7.66, 7.63, 7.58
5	CH	132.34	7.66, dd (8.5, 1.3)	7.63, 7.58, 7.54,	7.63, 7.58, 7.54
6	CH	131.49	7.58, d (8.3)	7.66, 7.54	7.66, 7.54
7	C	140.21	-		7.58, 7.07, 6.87
8/12	CH	130.55	7.07, d (8.2)	6.87	6.87
9/11	CH	126.56	6.87, d (8.2)	7.07, 5.51	7.07, 5.51
10	C	137.87	-		7.07, 6.87, 5.51
13	C	157.05	-		7.63
18	CH_2	50.12	5.51, s	6.87	6.87
20	C	153.52	-		5.51, 2.74, 1.61
22	C	158.33	-		1.59
23	C	118.42	-		5.51
24	CH_2	29.60	2.74, t (7.7)	1.61, 0.93	1.61, 0.93
25	CH_2	22.42	1.61, q (6.8)	2.74, 0.93	2.74, 0.93
26	CH_3	14.00	0.93, t (7.0)	2.74, 1.61	2.74, 1.61
27	C	71.63	-		1.59
28/29	CH_3	29.60	1.59, s		
31	C	162.24	-		5.02
34	CH_2	55.63	5.02, s		
35	C	142.23	-		5.02, 2.06
36	C	134.36	-		5.02, 2.06
38	C	153.43	-		
41	CH_3	9.10	2.06, s		

[a]Chemical shifts in ppm.

Table S2. ^{1}H, ^{13}C and 2D NMR data of **DP-1** in CD_3OD.

Position	Residue	$^{13}C^{a}$	$^{1}H^{a}$, multiplicity (J in Hz)	^{1}H-^{1}H COSY	^{1}H-^{13}C HMBC
1	C	141.02	-		7.63, 7.61, 7.54, 7.16
2	C	126.35	-		7.63, 7.56, 7.54
3	CH	131.69	7.63, dd (8.5, 1.6)	7.61, 7.56	7.61, 7.56
4	CH	128.86	7.56, m	7.63, 7.61, 7.54	7.63, 7.61, 7.54
5	CH	131.76	7.61, m	7.63, 7.56, 7.54	7.63, 7.56, 7.54
6	CH	131.81	7.54, m	7.61, 7.56	7.61, 7.56
7	C	140.78	-		7.54, 7.16, 7.06
8/12	CH	130.71	7.16, d (8.3)	7.06	7.06
9/11	CH	126.49	7.06, d (8.3)	7.16, 5.25	7.16, 5.25
10	C	136.39	-		7.16, 7.06, 5.25
13	C	158.58	-		7.63
18	CH_2	47.69	5.25, m	7.06	7.06
20	C	148.37	-		5.25, 2.63, 1.58
22	C	140.87	-		1.61
23	C	113.63	-		5.25
24	CH_2	29.83	2.63, m	1.58, 0.95,	1.58, 0.95
25	CH_2	22.65	1.58, sest (7.9)	2.63, 0.95	2.63, 0.95
26	CH_3	13.99	0.95, t (7.9)	2.63, 1.58	2.63, 1.58
27	C	70.28	-		1.61
28/29	CH_3	29.92	1.61, s		
31	C	158.45	-		

[a]Chemical shifts in ppm.

Table S3. ^{1}H, ^{13}C and 2D NMR data of **DP-2** in CD_3OD.

Position	Residue	$^{13}C^a$	$^{1}H^a$, multiplicity (J in Hz)	^{1}H-^{1}H COSY	^{1}H-^{13}C HMBC
1	C	142.82	-		7.57, 7.55, 7.12
2	C	127.80	-		7.47, 7.45
3	CH	131.72	7.55, d (7.4)	7.47	7.57, 7.47
4	CH	128.65	7.47, d (7.4)	7.57, 7.55	7.45
5	CH	131.26	7.57, m	7.47, 7.45	7.55, 7.47
6	CH	131.44	7.45, m	7.57	7.47
7	C	14145	-		7.45, 6.97
8/12	CH	130.74	7.12, d (8.3)	6.97	7.12, 6.97
9/11	CH	127.04	6.97, d (8.3)	7.12	7.12, 6.97
10	C	135.97	-		7.12, 5.38, 5.31
13	C	159.79	-		7.55
18	CH_2	49.01	5.38, d (16.8)<br>5.31, d (16.8)	5.31<br>5.38	6.97
20	C	147.64	-		5.38, 5.31, 4.24, 1.80, 1.64
22	C	125.62	-		
23	C	115.76	-		5.38, 5.31
24	CH_2	79.51	4.24, t (7.2)	1.80, 1.64	1.80, 1.64, 0.82
25	CH_2	27.84	1.80, m<br>1.64, m	4.24, 1.64, 0.82<br>4.24, 1.80, 0.82	4.24, 0.82
26	CH_3	10.29	0.82, t (7.4)	1.80, 1.64	4.24, 1.80, 1.64
27	OCH_3	57.06	3.08, s		4.24

[a]Chemical shifts in ppm.

Table S4. [1] H, ^{13}C and 2D NMR data of **DP-3** in CD_3OD.

Position	Residue	$^{13}C^a$	$^{1}H^a$, multiplicity (J in Hz)	^{1}H-^{1}H COSY	^{1}H-^{13}C HMBC
1	C	142.96	-		7.52, 7.49, 7.11
2	C	128.27	-		7.43
3	CH	131.79	7.52, dd (7.8, 1.6)	7.49, 7.43	7.49, 7.43
4	CH	130.26	7.43, dd (7.9, 1.5)	7.52, 7.49	7.52, 7.43
5	CH	131.16	7.49, t (7.8)	7.52, 7.43	7.52, 7.43
6	CH	128.27	7.43, d (8.0)	7.49	7.43
7	C	142.35	-		7.43, 7.11
8/12	CH	129.85	7.11, d (8.1)	7.29	7.29, 7.11
9/11	CH	130.54	7.29, d (8.1)	7.11	7.29, 7.11
10	C	135.74	-		7.11, 4.52, 4.43
13	C	162.08	-		7.52
18	CH_2	43.67	4.52, d (15.0)<br>4.43, d (15.0)	4.43<br>4.52	7.29
20	C	98.91	-		2.75, 1.87, 1.14, 0.90
22	C	161.38	-		
23	C	160.68	-		
24	CH_2	39.62	1.87, m	1.14, 0.90	1.14, 0.90, 0.72
25	CH_2	17.18	1.14, m<br>0.90, m	1.87, 0.90, 0.72<br>1.87, 1.14, 0.72	1.87, 0.72
26	CH_3	13.66	0.72, t (8.0)	1.14, 0.90	1.87, 1.14, 0.90
27	OCH_3	48.67	2.75, s		

[a]Chemical shifts in ppm.

Table S5. ^{1}H, ^{13}C and 2D NMR data of **DP-4** in CD_3OD.

Position	Residue	$^{13}C^{a}$	$^{1}H^{a}$, multiplicity (J in Hz)	^{1}H-^{1}H COSY	^{1}H-^{13}C HMBC
1	C	143.02	-		7.52, 7.49, 7.08
2	C	128.17	-		7.43, 7.42
3	CH	131.68	7.52, d (7.7)	7.42	7.49
4	CH	128.17	7.42, dt (7.5, 1.6)	7.52, 7.49	7.52, 7.49
5	CH	130.08	7.49, dt (7.5, 1.7)	7.43, 7.42	7.43, 7.42
6	CH	130.96	7.43, d (7.3)	7.49	7.49
7	C	142.39	-		7.43, 7.29
8/12	CH	130.96	7.08, d (8.3)	7.29	7.29, 7.08
9/11	CH	130.08	7.29, d (8.3)	7.08	7.29, 7.08
10	C	135.28	-		7.08
13	C	162.31	-		7.52
18	CH_2	43.90	4.84, overlapped<br>4.18, d (15.4)	4.18<br>4.84	7.29
20	C	100.16	-		4.84, 4.42, 4.18
22	C	160.01	-		
23	C	160.70	-		4.84, 4.18
24	CH_2	66.46	4.42, dd (11.2, 2.6)	2.16, 1.58	2.16, 1.58, 1.12
25	CH_2	26.67	2.16, dsest (7.5, 2.5)<br>1.58, m	4.42, 1.12	4.42, 1.12
26	CH_3	12.02	1.12, t (7.5)	2.16, 1.58	4.42, 2.16, 1.58

[a]Chemical shifts in ppm.

Table S6. [1] H, ^{13}C and 2D NMR data of **DP-5** in CD_3OD.

Position	Residue	$^{13}C^a$	$^1H^a$, multiplicity (J in Hz)	^1H-^1H COSY	^1H-^{13}C HMBC
1	C	142.86	-		7.61, 7.60
2	C	126.97	-		7.52, 7.48
3	CH	131.61	7.61, m	7.60, 7.52	7.60
4	CH	128.81	7.52, dt (8.1, 1.3)	7.61, 7.60	7.61, 7.48
5	CH	130.60	7.60, m	7.52, 7.48	7.61, 7.52
6	CH	131.69	7.48, dd (7.0, 1.8)	7.60, 7.52	7.52
7	C	141.69	-		7.48, 7.37
8/12	CH	130.66	7.13, d (8.1)	7.37	7.37, 7.13
9/11	CH	130.31	7.37, d (8.1)	7.13	7.37, 7.13
10	C	136.07	-		7.13, 4.65, 4.43
13	C	158.81	-		7.61
18	CH_2	44.08	4.65, x (xx)<br>4.43, x (xx)	4.43<br>4.65	7.37
20	C	99.30	-		4.07, 2.92, 1.45, 1.26
22	C	128.68	-		
23	C	160.68	-		
24	CH_2	66.88	4.07, x (xx)		1.45, 1.26, 0.74
25	CH_2	26.18	1.45, x (xx)<br>1.26, x (xx)	4.07, 1.26, 0.74<br>4.07, 1.45, 0.74	4.07, 0.74
26	CH_3	11.05	0.74, x (xx)	1.45, 1.26	4.07, 1.45, 1.26
27	OCH_3	48.80	2.92, s		

[a]Chemical shifts in ppm.

Table S7. ^{1}H, ^{13}C and 2D NMR data of **DP-6** in CD_3OD.

Position	Residue	$^{13}C^{a}$	$^{1}H^{a}$, multiplicity (J in Hz)	^{1}H-^{1}H COSY	^{1}H-^{13}C HMBC
1	C	142.90	-		7.20, 7.59, 7.51
2	C	128.72	-		7.51, 7.49, 7.59
3	CH	131.73	7.59, dd (7.6, 1.5)	7.49, 7.59	7.49, 7.59
4	CH	131.61	7.49, dt (7.6, 1.4)	7.59, 7.51	7.51, 7.59
5	CH	132.52	7.59, dt (8.4, 2.2)	7.49, 7.59, 7.51	7.59, 7.49, 7.51
6	CH	131.61	7.51, d (7.4)	7.49, 7.59	7.49, 7.59
7	C	141.35	-		7.26, 7.51, 7.20
8/12	CH	130.51	7.20, d (8.3)	7.26	7.26
9/11	CH	129.21	7.26, d (8.3)	4.62, 4.63, 7.20	4.62, 4.63, 7.20
10	C	135.84	-		7.20, 7.26, 4.62, 4.63
13	C	159.25	-		7.59
18	CH_2	44.00	4.63, d (14.7)<br>4.62, d (14.7)	7.26	7.26
20	C	174.25	-		4.62, 4.63, 1.84, 1.98, 4.91
21	CH	82.06	4.93, dd (6.5, 4.7)	1.84, 1.98, 0.93	1.84, 1.98, 0.93
23	C	157.25	-		4.91, 4.62, 4.63
25	CH_2	25.09	1.98, m<br>1.84, m	4.91, 0.93	4.91, 0.93
26	CH_3	8.39	0.93, t (7.5)	4.91, 1.84, 1.98	4.91, 1.84, 1.98

[a]Chemical shifts in ppm

Table S8. [1] H, ^{13}C and 2D NMR data of **DP-7** in CD_3OD.

Position	Residue	$^{13}C^{a}$	$^{1}H^{a}$, multiplicity (J in Hz)	^{1}H-^{1}H COSY	^{1}H-^{13}C HMBC
1	C	144.14	-		7.11, 7.66, 7.56
2	C	127.00	-		7.56, 7.66
3	CH	132.54	7.66, m	7.66, 7.56	7.66, 7.56
4	CH	129.02	7.56, m	7.56, 7.66	7.56, 7.66
5	CH	13163	7.66, m	7.66, 7.56	7.66, 7.56
6	CH	131.84	7.56, m	7.56, 7.66	7.56, 7.66
7	C	139.14	-		7.16, 7.56, 7.11
8/12	CH	130.51	7.11, d (8.4)	7.16	7.16
9/11	CH	127.26	7.16, d (8.4)	5.05, 7.11	5.05, 7.11
10	C	139.20	-		7.11, 5.05, 7.16
13	C	160.00	-		7.66
18	CH_2	47.01	5.05, s	7.16	7.16
20	C	178.71	-		5.05, 1.57, 2.48
21	CH_2	39.13	2.48, t (7.4)	0.86, 1.57	0.86, 1.57
22	CH_2	18.98	1.57, sest (7.2)	0.86, 2.48	0.86, 2.48
23	CH_3	13.79	0.86, t (7.6)	2.48, 1.57	2.48, 1.57
24	C	158.1	-		5.05

[a]Chemical shifts in ppm.

Table S9. [1] H, ^{13}C and 2D NMR data of **DP-8** in CD_3OD.

Position	Residue	$^{13}C^a$	$^1H^a$, multiplicity (J in Hz)	^1H-^1H COSY	^1H-^{13}C HMBC
1	C	141.75	-		7.06, 7.60, 7.62, 7.52
2	C	127.18	-		7.50, 7.52, 7.62
3	CH	130.32	7.62, d (7.5)	7.60, 7.50	7.60, 7.50
4	CH	130.22	7.50, dt (7.5, 1.4)	7.52, 7.60, 7.62	7.52, 7.60, 7.62
5	CH	130.19	7.60, dt (7.6, 1.5)	7.62, 7.50, 7.52	7.62, 7.50, 7.52
6	CH	130.32	7.52, dd (7.6, 2.0)	7.50, 7.60	7.50, 7.60
7	C	138.83	-		7.52, 7.18, 7.06
8/12	CH	128.85	7.06, d (8.4)	7.18	7.18
9/11	CH	126.84	7.18, d (8.4)	4.36, 7.06	4.36, 7.06
10	C	137.18	-		7.06, 4.36, 7.18
13	C	157.42	-		7.62
18	CH_2	41.63	4.36, s	7.18	7.18
20	C	160.96	-		4.36

[a]Chemical shifts in ppm.
